# Assembly and Development of the *Pseudomonas aeruginosa* Biofilm Matrix

**DOI:** 10.1371/journal.ppat.1000354

**Published:** 2009-03-27

**Authors:** Luyan Ma, Matthew Conover, Haiping Lu, Matthew R. Parsek, Kenneth Bayles, Daniel J. Wozniak

**Affiliations:** 1 Microbiology and Immunology, Wake Forest University Health Sciences, Winston-Salem, North Carolina, United States of America; 2 Department of Microbiology, University of Washington School of Medicine, Seattle, Washington, United States of America; 3 Pathology and Microbiology, University of Nebraska Medical Center, Omaha, Nebraska, United States of America; Massachusetts General Hospital, United States of America

## Abstract

Virtually all cells living in multicellular structures such as tissues and organs are encased in an extracellular matrix. One of the most important features of a biofilm is the extracellular polymeric substance that functions as a matrix, holding bacterial cells together. Yet very little is known about how the matrix forms or how matrix components encase bacteria during biofilm development. *Pseudomonas aeruginosa* forms environmentally and clinically relevant biofilms and is a paradigm organism for the study of biofilms. The extracellular polymeric substance of *P. aeruginosa* biofilms is an ill-defined mix of polysaccharides, nucleic acids, and proteins. Here, we directly visualize the product of the polysaccharide synthesis locus (Psl exopolysaccharide) at different stages of biofilm development. During attachment, Psl is anchored on the cell surface in a helical pattern. This promotes cell–cell interactions and assembly of a matrix, which holds bacteria in the biofilm and on the surface. Chemical dissociation of Psl from the bacterial surface disrupted the Psl matrix as well as the biofilm structure. During biofilm maturation, Psl accumulates on the periphery of 3-D-structured microcolonies, resulting in a Psl matrix-free cavity in the microcolony center. At the dispersion stage, swimming cells appear in this matrix cavity. Dead cells and extracellular DNA (eDNA) are also concentrated in the Psl matrix-free area. Deletion of genes that control cell death and autolysis affects the formation of the matrix cavity and microcolony dispersion. These data provide a mechanism for how *P. aeruginosa* builds a matrix and subsequently a cavity to free a portion of cells for seeding dispersal. Direct visualization reveals that Psl is a key scaffolding matrix component and opens up avenues for therapeutics of biofilm-related complications.

## Author Summary

*Pseudomonas aeruginosa* causes life-threatening, persistent infections in cystic fibrosis patients, despite highly aggressive antimicrobial therapy. Persistence is due, in part, to the ability of these bacteria to form surface-associated communities (biofilms) enmeshed in an extracellular matrix. This matrix is a poorly defined mixture of protein, polysaccharide, and DNA. An understanding of the organization and composition of the biofilm matrix will assist in the development of therapeutics aimed at disrupting biofilms. Using reagents that specifically recognize the *P. aeruginosa* Psl exopolysaccharide, we visualized matrix formation in real time during a biofilm development cycle. This revealed a highly organized and coordinated assembly of both polysaccharide and DNA components of the matrix. At late stages of biofilm morphogenesis, a Psl-free matrix cavity, occupied with numerous motile cells, developed. Mutants with reduced cell lysis were unable to form the Psl matrix cavity, whereas those with elevated cell death and lysis formed a larger matrix cavity, leading to accelerated dispersion. We propose that programmed cell death and autolysis are critical for the proper timing of biofilm development and dispersion. The data indicate that Psl is a key scaffolding component of the biofilm matrix, a property that likely plays a critical role in *P. aeruginosa* persistence.

## Introduction

Structured, surface-associated communities of bacteria (biofilms) are prevalent in environmental and clinical settings [1]. Biofilm bacteria are less susceptible to antimicrobial agents and are protected from the host immune response, giving rise to chronic infections that are notoriously difficult to eradicate [2],[3]. The extracellular polymeric substance (EPS) is thought to maintain the biofilm architecture and functions as a matrix, or glue, holding biofilm cells together and protecting them from shear forces in fluid environments [4]. By forming a matrix-encased multicellular aggregate, cells can also escape engulfment by phagocytic cells within a mammalian host. Surprisingly, little is known about how the extracellular matrix forms at distinct stages of biofilm development and which matrix components hold biofilm cells together on surfaces. A thorough understanding of the biofilm matrix ultra-structure is critical for the rational design of inhibitors that could prevent the numerous clinical and environmental complications associated with biofilms.

*Pseudomonas aeruginosa* is an opportunistic human pathogen that can cause life-threatening infections in cystic fibrosis (CF) patients and individuals with a compromised immune system [5]–[7]. This environmental bacterium can form biofilms on a variety of surfaces such as the mucus plugs of the CF lung, contaminated catheters, and contact lenses [8],[9]. *P. aeruginosa* has become a paradigm organism for biofilm research in the laboratory. Biofilms of *P. aeruginosa* develop in a five-stage muticellular cycle that is initiated by the attachment of free (planktonic) cells to a surface, followed by formation of microcolonies, and finally seeding dispersal, whereby swimming cells from microcolonies exit to occupy a new surface [4].

The matrix or EPS of *P. aeruginosa* biofilms is a poorly defined mix of polysaccharides, nucleic acids and proteins [10]–[13]. Exopolysaccharides, a main component of the extracellular matrix for animal and plant tissue, are also an important constituent of microbial biofilms [14]. At least three exopolysaccharides, Psl, Pel, and alginate contribute to biofilm formation in *P. aeruginosa* [11],[15]. The Psl exopolysaccharide, encoded from the polysaccharide synthesis locus (PA2231-2245), is required for bacterial cells to adhere to a substratum and maintaining biofilm structure [12], [16]–[18]. Our previous work showed that Psl appears to be composed of mannose, galactose, rhamnose, glucose, and trace amounts of xylose. However, the precise biochemical structure of Psl has not been determined. Although Psl was proposed to promote both cell-cell and cell-surface interactions, little is known about how Psl is anchored on the bacterial cell surface and how it might promote these interactions. To date, the exopolysaccharide matrix has not been directly visualized at distinct developmental stages during *P. aeruginosa* biofilm formation.

In addition to exopolysaccharides, extracellular DNA (eDNA) is an important component of the *P. aeruginosa* biofilm matrix [10],[12],[13]. The eDNA appears to be derived from random chromosomal DNA, which functions as a cell-to-cell inter-connecting component in the biofilm [10]. Cells also undergo autolysis in biofilm microcolonies [19], but it is unclear whether autolysis contributes to eDNA and biofilm development. It has also been shown that eDNA in the biofilm matrix contributes to cation gradients, genomic DNA release and inducible antibiotic resistance [20].

In the present report, we used Psl-specific lectin staining followed by confocal scanning laser microscopy (CLSM) to visualize Psl on the surface of cells and within the EPS matrix. The results show a helical pattern of Psl staining on the bacterial cell surface at the biofilm initiation stage. The anchoring of Psl on the cell surface is essential for formation of the Psl matrix and the initiation of a biofilm. The Psl matrix was visualized in real time during a biofilm developmental cycle. This reveals how the matrix forms and encases the bacterial cells in the biofilm and on a surface and how the matrix changes structure to maintain biofilm architecture during development. Using double staining methodologies, we show that the eDNA and Psl matrices do not overlap, but appear to coordinate activities to maintain the biofilm structure. We propose that, like higher organisms, *P. aeruginosa* can utilize programmed cell death and autolysis to degrade the Psl matrix in the center of microcolony to free ‘the seeds of biofilm’ for future dispersal.

## Results/Discussion

### Psl is anchored on the cell surface in a helical pattern

**Figure 1:**
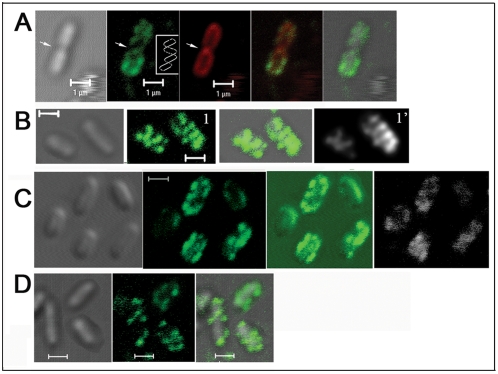
Lectin staining results of surface attached bacteria; Psl is anchored on the bacteria cell surface in a helical pattern. (A) MOA-FITC (green) and membrane stain FM4-64 (red) double staining of WFPA801 cells. The green image depicts the staining of helical structures around the cell surface as indicated in the inset. The white arrow points out the division site. (B) An optical section of HHA-FITC stained WFPA801 cells without (1) or with (1') deconvolution. (C) HHA-FITC and MOA-TRITC (white) double staining of WFPA801 cells. (D) HHA-FITC stained PAO1 cells. In all panels, green and red signals are merged images of the FITC-lectins and FM4-64 stained cells. The gray signal represents DIC images while green with gray are merged images of the FITC-lectins with DIC images. Scale bars, 1 µm.

To visualize Psl on the bacterial cell surface and to detect Psl exopolysaccharide during the attachment stage of biofilm development, we used fluorescently labeled lectins MOA (from *Marasmium oreades* agglutinin) or HHA (from *Hippeastrum hybrid*) to stain individual *P. aeruginosa* cells. The lectins MOA and HHA detect the galactose and mannose structure in Psl, respectively, and we previously demonstrated that they specifically stained Psl [21]. The cells were allowed to attach to a glass surface keeping Psl in its native state on the bacterial surface. When surface-attached *P. aeruginosa* WFPA801 (Psl-overproducing strain) cells were stained by FITC-MOA, we found that Psl was associated with the bacterial cell surface. More interestingly, most cells had a helical-like patterned fluorescence signal (green in Figure 1A). Such patterns were only seen with the MOA stained Psl and not with FM4-64 (red in Figure 1A) that stained the cell membrane. These helical patterns were observed on dividing (Figure 1A) as well as non-dividing cells (Figure 1B and 1C). The helical pattern of Psl on the cell surface was also seen with HHA staining (green, Figure 1B). Moreover, FITC-HHA and TRITC-MOA double staining of WFPA801 cells showed a similar helical pattern (compare green with white images in Figure 1C).

In raw images, the helical structures appeared as a series of poorly resolved diagonal strips (image 1, Figure 1B). To obtain deconvoluted images [22]–[24], we optically sectioned individual cells to a series of images whose focal planes extended across the cell width. Most out-of-focus information was removed from each section by iterative deconvolution to gain sharper images. After deconvolution, the signal appeared as a series of parallel curved diagonal bands that represented one face of a helical array that coiled around the cell (image 1', Figure 1B).

The helical pattern of Psl staining in Figure 1A–1D was observed in WFPA801 cells grown under conditions that express elevated levels of Psl [18]. To verify that the helical pattern was not an artifact of Psl overproduction, we stained surface-attached wild type PAO1 cells. Although more difficult to discern, Psl also had a helix-like pattern on the PAO1 cell surface (green in Figure 1D).

Cell-cell interactions are a basic element of a biofilm structure. Previous data suggest that Psl promotes cell-cell interactions [12], [16]–[18]. The helical distribution of Psl on the surface of *P. aeruginosa* may readily promote interactions with Psl on adjacent bacteria. This will establish a matrix and may enhance the cell-cell interactions since contacts between adjacent helices often stabilize macromolecules. Moreover, a helical distribution may be a property conserved among rod-shaped bacteria, allowing them to efficiently organize their cell periphery. Bacterial cytoskeleton proteins such as actin-related protein MreB, tubulin-like protein FtsZ, and division site placement proteins MinCDE form helical filaments coiled around the rod shaped bacteria cell [22]–[24]. Outer membrane proteins and lipopolysaccharides also have a helical pattern anchored on the cell surface [25], and a helical mechanism of peptidoglycan assembly has also been reported [26]. The Psl polysaccharide itself may exhibit a helical structure, since intertwined helices can also be formed between polysaccharide polymers [27].

The most logical explanation for the helical pattern of Psl is that the Psl biosynthesis machinery is itself organized in such a pattern. This would allow for coupled synthesis, export, and assembly of the cell surface-associated Psl. Alternatively, outer membrane components such as proteins or lipids that have a helical mode of insertion in the outer membrane may be anchoring Psl.

**Figure 2:**
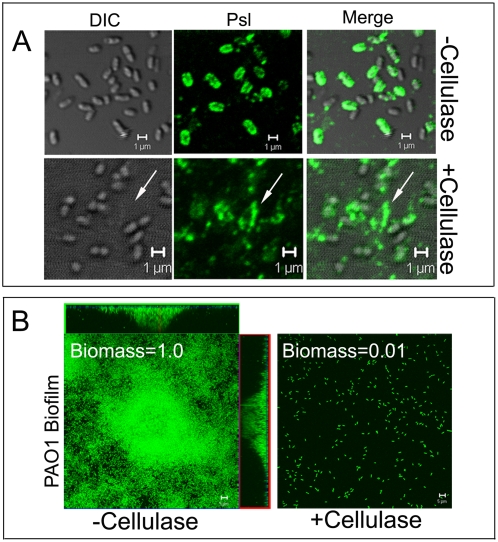
The effect of cellulase treatment on Psl localization and biofilm formation. (A) HHA-FITC (green) staining of surface-attached WFPA801 cells with or without cellulase treatment. (B) The biofilms of GFP-tagged PAO1 grown in flow cells in the presence or absence of cellulase. The total biomass was quantified by COMSTAT software. Values shown on the upper left corner of the corresponding image have been normalized to the result of the non-cellulase treatment sample (3.1 µm^3^/µm^2^). The arrows reveal Psl that has dissociated from the bacterial surface and adhered with the substratum. Scale bars, 1 µm for panel A and 5 µm for panel B.

To investigate whether surface association and the helical localization pattern of Psl contribute to biofilm formation, we utilized cellulase, which targets β-1,3 or β-1,4-linked glucans [28]. Previously, chemical composition analyses and Congo Red staining [18],[21],[29] showed that the Psl exopolysaccharide contains β-1,3 or β-1,4-linked glucose. Cellulase readily hydrolyzed Psl and eliminated the helical distribution of Psl from the cell surface (Figure 2A). Without cellulase, most cells had Psl associated with the cell surface. With cellulase treatment, Psl appeared to dissociate from the bacterial surface, even though the overall fluorescence intensity was similar between cellulase-treated and untreated samples. Moreover, when Psl dissociated from the bacterial surface it appeared to attach to the glass cover slip (white arrow, Figure 2A). Surprisingly, crystal violet attachment assays [30] showed that overnight cellulase-treated PAO1 cells were able to attach as well as untreated cells (data not shown). These data indicate that cellulase can free Psl from the bacterial cell surface without degrading the major Psl polymer and cellulase-digested Psl is able to adhere with a substratum. This data also suggests that β-1, 3 or β-1,4-linked glucose may be the monosaccharide that links Psl to the bacterial surface.

To test the effect of cellulase on biofilm formation under flow conditions, we grew a GFP-tagged PAO1 strain in the absence or continued presence of cellulase. Under these conditions, cellulase treatment dramatically reduced biofilm development (Figure 2B). This biofilm phenotype is similar to that observed with the *Δpsl* strain [18]. COMSTAT analysis [31] showed that the total surface-bound biomass of cellulase-treated PAO1 was only 1% compared with similarly grown non-treated PAO1. This difference was not due to growth inhibition since PAO1 grown in the presence or absence of cellulase had similar growth rates (data not shown). We cannot exclude the possibility that cellulase is indirectly affecting Psl cell surface association, such as targeting one or more β-1,3 or β-1,4-linked glucans that associate with Psl. Irrespective of the mechanism, these data reveal that the surface association of Psl is essential for *P. aeruginosa* to initiate a biofilm.

### At an early stage of biofilm development, Psl forms a matrix holding bacteria cells in the biofilm and on the surface

**Figure 3:**
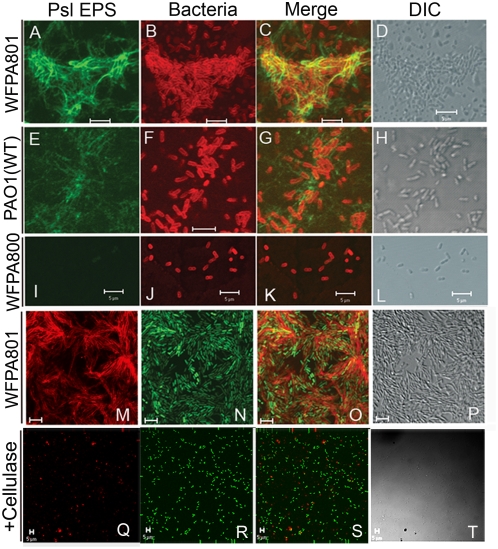
Psl at an early stage of biofilm development: a matrix formed by Psl holding bacterial cells in the biofilm and on the surface. (A–D) Staining of Psl matrix in biofilms formed by strains PAO1, WFPA801, and WFPA800: images were acquired after 20 hours in Jensen's media under continuous flow conditions with 2% arabinose. Biofilms in reactors were stained for 2 hours with lectins as follows: HHA-FITC and FM4-64 stained WFPA801 biofilm. (E–H) HHA-FITC and FM4-64 stained PAO1 biofilm. (I–L) HHA-FITC and FM4-64 stained WFPA800 biofilm. (M–P) MOA-TRITC staining of GFP-tagged WFPA801 biofilm. (Q–T) MOA-TRITC staining of GFP-tagged PAO1 biofilm, grown under flow conditions in Jensen's media with cellulase. Scale bars, 5 µm.

To detect the Psl matrix of biofilms at early stages of development, we used fluorescently labeled Psl lectins to stain live biofilms grown in flow cell chambers (Figure 3A, 3E, 3I, 3M). Wild type PAO1, Psl-overproducing strain WFPA801, and *psl* mutant strain WFPA800 were evaluated. Bacteria were counter-stained red using the membrane stain FM4-64 (Figure 3B, 3F, 3J) or tagged with GFP (Figure 3N, 3R). Psl was readily visualized using HHA-FITC (green) or with MOA-TRITC (red) and formed a matrix in WFPA801 and PAO1 (green in Figure 3A, 3E and red in Figure 3M) but not in cells of the *Δpsl* strain WFPA800 (Figure 3I). In most cases, Psl matrix material was associated with the bacteria cells, which can be observed in the FITC-HHA stained WFPA801 biofilm (Figure 3A–3C) and in the TRITC-MOA stained matrix (Figure 3M–3O). Interestingly, Psl matrix material was also detected in areas that had no bacteria cells (Figure 3C, 3G, and data not shown). It is not clear if this Psl has been released from cells or whether it remains attached on the cell surface. This free Psl matrix material may promote attachment on surfaces or play a role in recruiting planktonic bacteria to the biofilm. The above results also provide evidence that Psl promotes cell-cell and cell-surface interactions. This occurs by Psl facilitating adherence to surfaces (Figure 3A, 3E and 3M), surrounding cells, connecting cells together, and recruiting cells to the surface (Figure 3C, 3G, 3O). The net result is formation of a matrix, which holds bacterial cells in the biofilm and on the surface.

In order to determine if the Psl matrix can form with continued cellulase treatment, MOA-TRITC was used to stain the GFP-tagged PAO1 biofilm grown in the presence of cellulase under flow conditions for 20 hours. PAO1 was severely compromised in its ability to form the Psl matrix (Figure 3Q), which is consistent with the biofilm phenotype under these conditions (Figure 2B and Figure 3Q). This indicated that a biofilm was not able to develop if matrix formation was inhibited. As cellulase dissociated Psl from the bacterial surface and eliminated the helical distribution of Psl, these data strongly suggest that the cell surface anchoring property of Psl is necessary for Psl to promote cell-cell interactions and this interaction is critical for Psl to initiate a matrix under flow conditions.

### How the Psl matrix maintains the biofilm architecture

**Figure 4:**
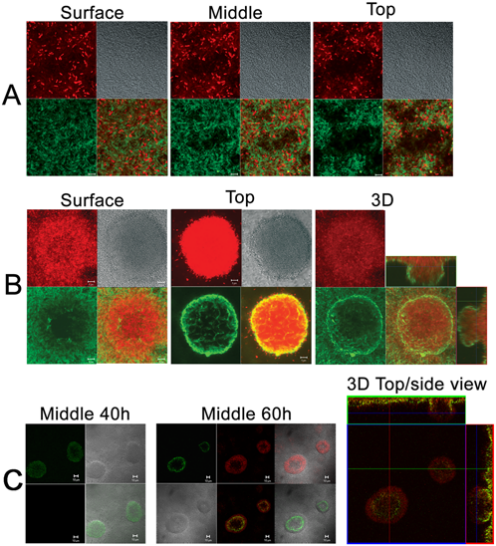
How the Psl matrix maintains the biofilm architecture. Shown are sets of optical sectioned images acquired at different locations of the biofilm (position indicated on the top of each panel). DIC images are in gray. Green and red merged images are shown at the lower right corner for panels A and B. Bar, 5 µm for panels A and B, 10 µm for panels C and D. (A) Psl matrix (green, MOA-FITC staining, bottom left) in a multilayer biofilm (4 µm thickness) of WFPA801 (red, FM4-64 staining, upper left). (B) Psl matrix (green, MOA-FITC staining, bottom left) in a WFPA801 biofilm microcolony (FM4-64 staining, 24 µm thickness, upper left). The top-down view (square) and side view (rectangle) of 3D reconstituted images are shown, which reveals how the peripherally localized Psl matrix encases the bacteria in a mushroom-like microcolony. (C) The newly synthesized Psl matrix (red) covers the existing Psl matrix (green). The Psl matrix of WFPA801 biofilms was stained with MOA-FITC (green) at 40-h-growth and stained again by MOA-TRITC (red) at 60-h-growth. The large square image is a horizontal section at the top of microcolony. The blue line in the side view images marks the location of the section (rectangle). Green and gray merged images are at the lower right. Green and red merged images are at the right panel and the bottom middle of the middle panel.

Depending on conditions, biofilms can form either flat multilayer structures or microcolonies that have defined 3-dimensional arrangements. To visualize how the Psl matrix maintains the biofilm architecture, we studied the distribution of Psl in these two types of communities by optically sectioning MOA-stained biofilms. In a flat biofilm of WFPA801, Psl matrix was equally distributed and associated with bacterial cells (Figure 4A). However in a well-defined 3D microcolony structure, the Psl matrix was unevenly distributed. This was visualized in representative horizontal Z-images of a microcolony (Figure 4B, left and middle panels), a 3-D reconstruction of the microcolony (Figure 4B, right panel) or a series of 1 µm Z-stacks of the microcolony reconstructed into a movie file (Video S1). Here, enhanced lectin staining was observed in the periphery of each microcolony and reduced lectin staining was seen in the center of microcolonies (middle panel in Figure 4B). In the mushroom-like microcolonies, little Psl staining was detected in the lower center (the area from the microcolony center to the surface area, left panel of Figure 4B). This staining pattern resulted in a matrix-free cavity in the lower center of the mushroom-like microcolony. In some cases, such as that seen in the microcolony close to the substratum, there were fewer bacteria in the center than in the microcolony periphery (left panel, Figure 4B). However, at the top of the microcolony there was little difference in the density of cells in the center versus those in the periphery (Figure 4B, middle panel). This was observed by staining the microcolonies with either FM4-64 (Figure 4B) or when the cells expressed GFP (Video S1). A similar matrix distribution pattern was observed using biofilm-grown wild type PAO1, indicating the Psl staining pattern in biofilms was not due to Psl overproduction (data not shown). No Psl was detected in either multiple layer biofilms or microcolonies formed by the *Δpsl* strain WFPA800 (data not shown). Overall, our data shows that Psl surrounds the constituent cells in either a multilayer biofilm or a 3D-structured microcolony (see 3D view of the microcolony in the right panel of Figure 4B and the series of Z-stacks in Video S1).

Although some microcolonies did reveal limited Psl staining in the center of the Psl matrix cavity, most Psl staining was peripheral. To verify that the Psl matrix cavity in the center of the microcolony is not an artifact of incomplete lectin diffusion, we increased the lectin concentration 5-fold and doubled the incubation time. However, the staining pattern did not change and the matrix cavity was still observed (data not shown). To determine if the lectin size (∼50 kDa) is a limiting factor in diffusion into the microcolony, we used 70 kDa FITC labeled dextran beads to stain microcolonies. The dextran beads readily penetrated the lower center of microcolonies (data not shown). Therefore, the appearance of the Psl matrix cavity is likely due to reduced Psl in the center of the microcolony and not insufficient lectin penetration.

To investigate where newly synthesized Psl is located in the microcolony and how existing Psl matrix might change during biofilm growth, we pulse stained biofilms with MOA-FITC (green) at 40 hours growth followed by MOA-TRITC (red) staining at 60 hours growth. At the 40-hour time point, there was considerable Psl staining in the center of microcolonies (green stain Figure 4C, left panel). At the 60-hour time point, most Psl staining was peripherally localized. The newly synthesized Psl (Figure 4C, red) covered the pre-existing Psl matrix (Figure 4C, green) and there was little newly synthesized Psl staining in the center of the microcolony. These data suggest that bacteria in the center of the microcolony do not continually produce Psl. Instead, pre-existing Psl accumulates at its original peripheral location, indicating that Psl degradation may occur in the center of microcolonies. Since bacteria in the periphery actively synthesize Psl, peripheral accumulation of Psl may be due to interactions of cell-associated Psl with free Psl in the matrix. The net result is a Psl matrix-free area in the center of the microcolony (Figure 4C, compare the green images in the left panel with the middle panel).

The peripheral localization of Psl may be important in maintaining the 3-dimensional structure of the microcolony as well as providing surface adherence. The peripheral Psl may also function in recruiting free-swimming planktonic bacteria to the microcolony (e.g. the merged image in Figure 4B, middle panel). The peripheral staining of Psl in the microcolony does not appear to be the result of differences in *psl* transcription, since both wild type PAO1 and the Psl-inducible strain WFPA801 showed a similar Psl distribution pattern. Bacteria in the center of the microcolony may produce less Psl due to the lack of nutrients or through a posttranscriptional control mechanism. The level of intracellular cyclic-di-guanylate controls the production of Psl and Pel exopolysaccharide [32],[33]. Perhaps nutrient gradients in the microcolony [34],[35] lead to gradients of intracellular cyclic-di-guanylate from the periphery (highest) to the center of the microcolony (lowest), which may explain the changes in Psl localization. In *Staphylococcus and P. aeruginosa*, active DNA replication and protein synthesis were observed in the periphery of biofilm microcolonies [34],[36]. Thus, enhanced metabolic activity may also be one of factors that result in the Psl peripheral localization in the microcolony. Alternatively, enzymes released from bacteria in the microcolony center may degrade the pre-existing Psl matrix.

### The Psl matrix cavity prepares biofilms for future seeding dispersal

**Figure 5:**
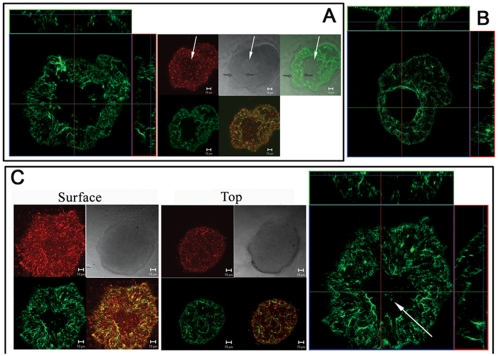
The Psl matrix of microcolonies before and after dispersion. Panels A–C show a 2-day-old PAO1 biofilm stained by HHA-FITC (green) and FM4-64 (red). Bar, 10 µm. (A) The Psl matrix of a microcolony undergoing dispersion (28 µm thickness). On the left is a horizontal sectioned image of the matrix (square) close to the surface and two vertical sectioned images of the matrix (rectangle). The small squares on the right show horizontal sectioned images from the middle of the same microcolony. The white arrows point out the areas with swimming dispersing cells and the two black arrows indicate the immobile bacterial wall. The merged image reveals how the Psl matrix enmeshed the bacteria in the immobile wall and covers dispersing cells in the matrix cavity. (B) The Psl matrix of a microcolony after seeding dispersal. A horizontal sectioned image (square) of the microcolony near the top and two vertical sectioned images (rectangle) are shown. (C) A Psl matrix cavity in a microcolony (33 µm thickness) with no visible swimming cells. Shown on the right is a horizontal sectioned image of the matrix close to the surface (square) and two vertical sectioned images of the matrix (rectangle). The large white arrow points to the Psl staining in the center of a Psl matrix cavity. Two sets of images sectioned at the top of the microcolony (middle panel) or close to the surface (left panel) are shown.

We next performed lectin staining on microcolonies at the dispersion stage of biofilm development. Active dispersion is indicated by the presence of numerous swimming bacteria in the center of aged microcolonies (see Video S2). When PAO1 biofilms were stained with both HHA-FITC and FM4-64, the Psl matrix enmeshed the bacteria in the immobile cluster wall (Figure 5A, area between the black arrows), which surrounded an area with swimming cells undergoing seeding dispersal (Figure 5A, white arrows). Little Psl matrix was detected in the region with swimming cells located at the lower center of the microcolony (Figure 5A, and Video S2). A Psl matrix cavity was also present in the center of a PAO1 microcolony with no visible swimming cells (Figure 5C) as we observed in the microcolony of WFPA801 (Figure 4B). This suggests that the Psl matrix cavity in the microcolony center has to be prepared prior to the appearance of swimming dispersing cells. The Psl matrix of a microcolony after dispersion showed an empty hole in the center with the remaining matrix intact (Figure 5B).

### Cell death and lysis contributes to the Psl matrix cavity formation and biofilm development

**Figure 6:**
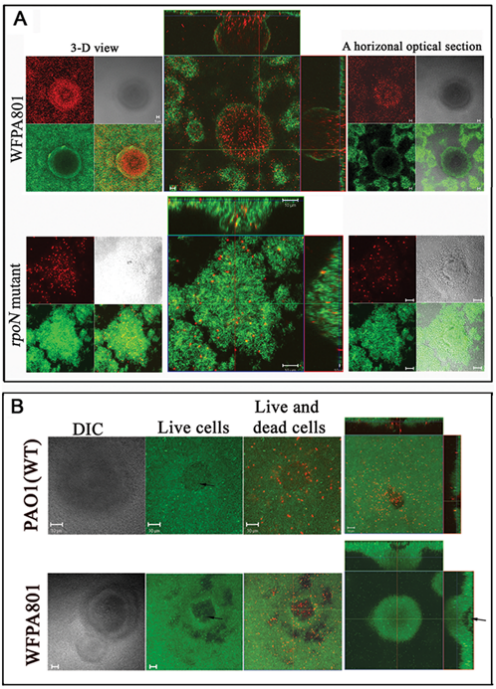
Cell death and lysis contributes to the Psl matrix cavity formation. In panel A, Psl matrix was stained in green, whereas in panel B, the green fluorescent signal represents viable cells stained by SYTO9. In all panels, red fluorescence is due to propidium iodide (PI) staining of either eDNA (weak and diffuse red) or cells with a compromised cell membrane (dead cells, bright concentrated red). Bar, 5 µm for WFPA801 in panel A and 10 µm for the other images. (A) Images of 2-day-old WFPA801 and *rpoN* mutant biofilms stained by HHA-FITC (green) and PI (red). A top-down view of 3D reconstructed images is shown in left panel. Sets of images optically sectioned (horizontally) at the neck of the same microcolony are shown in the right panel. The corresponding merge image of Psl matrix and DNA matrix is shown in the middle panel (large square). Two corresponding vertical section images are also shown (rectangle). (B) LIVE/DEAD viability staining of PAO1 and WFPA801 biofilm microcolonies show that there are viable swimming cells in the lower center of the microcolony and dead cells/extracellular DNA that fill up all void spaces. Three microcolonies prior to dispersion and one microcolony (the upper right) post the seeding dispersal are shown. The arrow points out the swimming cells in the center of the microcolony.

The *P. aeruginosa* biofilm matrix has a considerable amount of extracellular DNA (eDNA [10],[12],[13]. Cell autolysis, which occurs in microcolonies, is believed to mediate eDNA release [19]. To investigate where Psl and eDNA localize in the biofilm matrix, we performed HHA-FITC lectin and propidium iodide (PI) staining (Figure 6A). PI will stain eDNA or DNA in cells with a compromised cell membrane. The results showed that there was little overlap between Psl (green) and eDNA (diffuse red) or membrane permeable cells (Figure 6A, top panel, bright red; hereafter these cells were named “dead”). In the 3D microcolonies of WFPA801 (Figure 6A), the dead cells and eDNA were mostly located in the area that had little Psl matrix. Interestingly, the dead cells were concentrated in a region close to the substratum and in the stalk portions of mushroom-like microcolonies, which was also the location of the matrix cavity. This is most evident in the reconstructed side view images (rectangles in the center of Figure 6A).

To study whether cell death in the microcolony is correlated with the Psl matrix cavity formation, we stained the dead cells and Psl matrix of biofilms formed by *rpoN* mutants. These strains have little cell death in the biofilm microcolony [19] and this was verified in our analysis (Figure 6A, lower panel). In contrast with what we observed with wild type *P. aeruginosa*, Psl was equally distributed in the center and periphery of microcolonies of the *rpoN* mutant (Figure 6A). Similar findings were seen with a *fliM pilA* mutant, which also had reduced cell death in the biofilm microcolony ([19], and data not shown). We also performed Live/Dead staining on WFPA801 and PAO1 biofilms grown under the same conditions as those in Figure 6A. We found numerous microcolonies at the stage prior to dispersion, since they had swimming cells in the microcolony center (see Video S3). Interestingly, the dead cells and eDNA filled up the void spaces inside microcolonies and surrounded the swimming cells (Figure 6B, first three images). Numerous dead cells were found in the void space of the microcolony post seeding dispersal (Figure 6B, the image at upper right corner). The space with swimming cells was located in the same region with little detectable Psl (Figure 6B, the image at lower right corner).

Prior studies revealed that DNase treatment disrupts *P. aeruginosa* biofilm structural integrity [19]. Dead cells, which have a compromised cell membrane, eventually release DNA that could be incorporated into the eDNA matrix or accelerate cell death and lysis [20]. Our data indicate that, while the Psl and DNA components of the matrix do not overlap, Psl and eDNA may function cooperatively to encase the bacteria in the biofilm. Interestingly, the region in the microcolony that has little Psl is in the same location with concentrated dead cells and subsequently, swimming cells at the dispersion stage. Moreover, mutants that have reduced cell death do not form a Psl matrix cavity. This data suggests that cell death and lysis are responsible for forming the matrix cavity in the microcolony.

### The *P. aeruginosa cidAB* and *lrgAB* genes control cell death and lysis, as well as the timing of seeding dispersal

**Figure 7:**
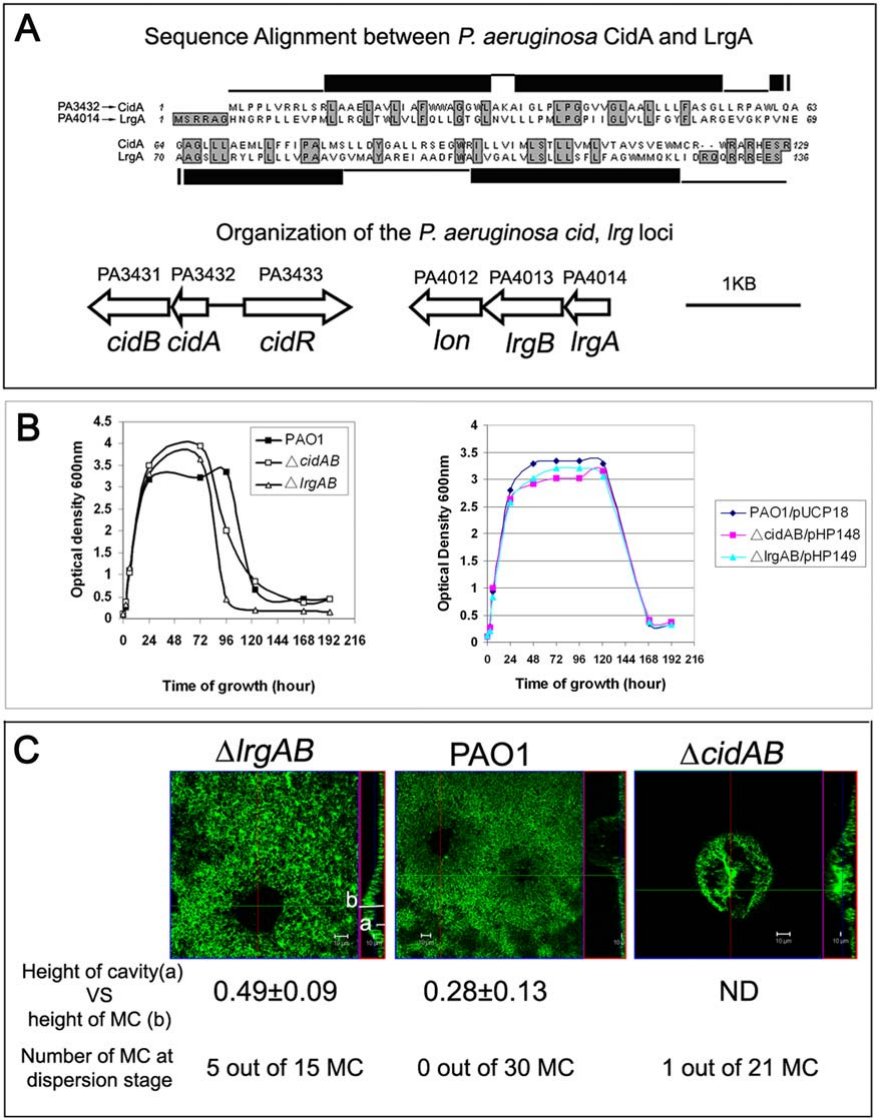
The *P. aeruginosa cidAB* and *lrgAB* genes control cell death and lysis, as well as the timing of seeding dispersal. (A) A sequence alignment between *P. aeruginosa* CidA (putative holin) and LrgA (putative anti-holin), the predicted CidA protein structure, and a diagram of *P. aeruginosa cid*/*lrg* genetic organization. Identical residues between the two sequences and residues absent in CidA are shaded. The black rectangle represents a sequence predicted to form a trans-membrane helix. _, residues predicted to be cytoplasmic; -, residues predicted to be periplasmic. (B) A growth comparison of *P. aeruginosa* PAO1, Δ*cidAB*, and Δ*lrgAB* (left graph) and the corresponding complemented strains (right graph). (C) The Psl matrix (green, HHA-FITC staining) cavity in the 2-day-old biofilm of Δ*lrgAB*, PAO1, and the Δ*cidAB* mutant. A horizontal sectioned image (square) and a vertical sectioned image (rectangle) are shown. The white bar marked by letter “a” represents the height of the Psl matrix cavity and the “b” bar shows the height of the corresponding microcolony (MC).

Our results (Figure 6) as well as other reports [10],[20],[37], indicate that death and lysis of cells occurs as a function of their spatial orientation within the biofilm, like that recently reported in *S. aureus* [38]–[40]. This suggests that *P. aeruginosa* is capable of undergoing a form of programmed cell death similar to apoptosis in higher organisms. Previous studies with *S. aureus* show that the CidA and LrgA proteins function as a holin and anti-holin, respectively, to control cell death and the timing of cell lysis [40]. The holins are phage-encoded small integral membrane proteins that control the activity of murein hydrolases and timing of host cell lysis during bacteriophage infection, whereas the anti-holin molecule antagonizes holin activity [40]. Holins are the gatekeeper of the lysis process and at a precise time point, can form large holes in the cytoplasmic membrane of phage-infected bacteria [41]. *cid/lrg* orthologues are present in a variety of bacteria including *P. aeruginosa* [38]. Based on structural analysis, sequence homology, and gene organization, we have identified a putative *cidAB* (PA3432-3431) and *lrgAB* (PA4014-4013) locus in *P. aeruginosa* (Figure 7A). The PA3432 encoded protein was tentatively identified as CidA, since it had all the structural features of a holin. These include a small trans-membrane protein (129 amino acids, four predicted trans-membrane helices as shown by black rectangles in Figure 7A), a hydrophobic N-terminus, and a highly polar, charged C-terminal domain. PA4014 was defined to encode LrgA, which has characteristics of an anti-holin protein (Figure 7A). As with *S. aureus*, LrgA shares sequence similarity with CidA, but LrgA has an extended N-terminus including two positively charged residues (arginine), which are important for anti-holin function [42]. Similar to the organization in *S. aureus*, the *P. aeruginosa cidA*/*lrgA* orthologues had *cidB*/*lrgB*, respectively, immediately downstream, and upstream of *cidA* is a putative transcriptional regulator (*cidR*).

To investigate whether the putative Cid/Lrg system controls cell death and lysis in *P. aeruginosa*, we constructed *cidAB* and *lrgAB* in frame deletion strains. During the 24–72 hour growth period in liquid culture, both mutants exhibited higher OD_600_ values than the parental strain PAO1 (Figure 7B, left graph). However, both mutants had a similar number of viable cells as PAO1 at these time points (based on CFU determination; data not shown). This is similar to the phenotype of *cidA*/*lrgA* mutants in *S. aureus* [39]. Deletion of either *cidAB* or *lrgAB* appeared to cause increased cell death resulting in the accumulation of dead cells and hence, increased OD_600_ values. More interestingly, cell lysis occurred earlier in the Δ*cidAB* and Δ*lrgAB* mutants than in PAO1 (Figure 7B, left graph). Compared with PAO1 and the Δ*lrgAB* mutant, the Δ*cidAB* mutant appeared to undergo lysis at a slower rate (Figure 7B, left graph). Consistent with this phenotype, Live/Dead staining of biofilms showed that the Δ*cidAB* mutant accumulated more dead cells in the biofilm than PAO1 (data not shown). The Δ*cidAB* and Δ*lrgAB* complemented strains had cell lysis similar to that observed with PAO1 (Figure 7B, right graph). Collectively, these results suggest that the putative Cid/Lrg system controls cell death and timing of cell lysis in *P. aeruginosa*.

To determine if the Cid/Lrg system affects *P. aeruginosa* biofilm development, we examined biofilms of parental wild type, Δ*cidAB*, and Δ*lrgAB* mutants. At the time point chosen, none of the microcolonies formed by the wild type strain and only one from the Δ*cidAB* mutant had undergone dispersion (Figure 7C). However, one-third of the Δ*lrgAB* microcolonies were undergoing active dispersion. Consistent with these biofilm development effects, the microcolonies of the Δ*lrgAB* mutant formed a larger Psl matrix cavity than PAO1 (Figure 7C). This was evaluated by calculating the ratio of the matrix cavity height (white bar, letter a in Figure 7C) by the height of the microcolony (white bar, letter b in Figure 7C; 0.49±0.09 for the Δ*lrgAB* mutant and 0.28±0.13 for the parental PAO1 strain). The data was calculated from 15 independent microcolonies and the difference between Δ*lrgAB* and PAO1 was statistically significant (P<0.001, 2 tail t-test). In the Δ*cidAB* mutant, there was no clearly defined matrix cavity. Instead, Psl accumulated in the center of microcolony (Figure 7C, right panel). These data were consistent with the behavior of Δ*cidAB* and Δ*lrgAB* mutants in liquid media. Compared with the parental PAO1 strain, the Δ*cidAB* mutant had more cell death but reduced cell autolysis, which resulted in Psl accumulation in the center of the microcolony. The Δ*lrgAB* mutant also had more cell death but a normal cell lysis rate. This led to a larger matrix cavity and premature seeding dispersal. Overall, the above results showed that programmed cell death and lysis contribute to the formation of the Psl matrix cavity and seeding dispersal, suggesting that cell autolysis was important for Psl degradation and localization in the microcolony.

Collectively, we propose that *P. aeruginosa* utilizes at least two mechanisms to generate a Psl matrix-free area in the center of microcolonies. First, the bacteria in the center of the microcolony reduce Psl synthesis and second, a subset of bacteria undergo cell death and lysis, releasing bacterial surface-bound Psl, eDNA, and perhaps enzymes that may degrade Psl in the center and clear an area for the future dispersing cells. Psl degradation may also be enhanced by the recently described mechanism of eDNA cation sequestration in *P. aeruginosa* biofilms [20].

### Conclusions

**Figure 8:**
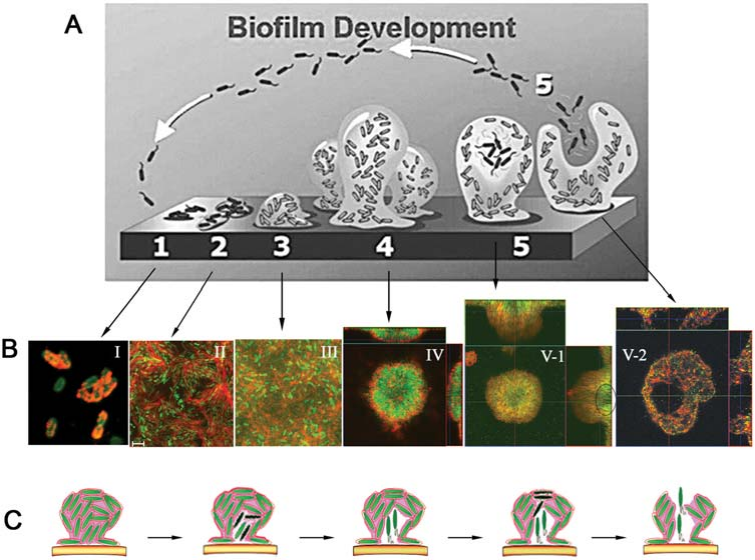
How the *P. aeruginosa* Psl biofilm matrix forms and develops. (A) A schematic showing five stages of biofilm development. Created by P. Dirckx, K. Sauer, and D. Davies and used with permission of the authors [4] and the *Annual Review of Microbiology*, Volume 56 ©2002 by Annual Reviews (www.annualreviews.org). (B) Selected images of Psl staining (red) during each development stage of biofilm formation. Green fluorescence signal is derived from GFP-labeled *P. aeruginosa*. The circle in the image V-I depicts the Psl matrix cavity. (C) A proposed mechanism showing how a *P. aeruginosa* microcolony sacrifices a portion of cells in the center and disrupts the existing matrix to free cells for dispersion. The pink material represents the Psl matrix, the light green cells represent live bacteria, and the dark green cells represent dead bacteria undergoing autolysis.

A representative image of the Psl matrix at each biofilm development stage is shown in Figure 8B. Overall, the Psl matrix remains intact at all stages of biofilm development to facilitate surface adherence and maintain biofilm architecture. At early developmental time-points, Psl (red) associates predominately with the cells (green) on the surface (Figure 8B, stage I). The cell-associated Psl connects bacteria with each other, encasing each bacterium within a matrix (Figure 8B, stage II). This appears to be promoted by the helical distribution of Psl on individual cells. Upon further development, multiple layers of cell aggregates form. In such biofilms, Psl covers the entire structure from top to bottom (Figure 8B, stage III). Once a biofilm progresses to a 3-dimensional microcolony, Psl is observed in the periphery rather than the center of the microcolony (Figure 8B, stage-IV). During this maturation, a matrix cavity is formed in the lower center of the mushroom-like microcolony (Figure 8B, stage-V-1), which prepares the biofilm for future dispersion events. The matrix cavity and swimming dispersing cells are located in the region close to the substratum and in the stalk of the mushroom-like microcolony. After seeding dispersal, a large gap is left in the center of the matrix, but the remainder of the microcolony is intact (Figure 8B, stage-V-2). The location of the matrix cavity and dispersing cells in our study (Figure 6B, Figure 8B) is at the substratum, which differs from that described by others who used light microscopic techniques (Figure 8A) [4]. It must be emphasized that the results obtained with our experimental system may be specific to *P. aeruginosa* and without supporting data may not be extrapolated to other systems.

We propose a mechanism for how *P. aeruginosa* utilizes programmed cell death and lysis to make a matrix cavity to free a portion of cells for future dispersion (Figure 8C). A microcolony sacrifices a portion of cells, which undergo autolysis in the center (dark green cells in Figure 8C). This will release the Psl that is associated with the surface of these cells and disrupt the existing matrix. Cell lysis will also liberate eDNA, nutrients, and enzymes, which can degrade matrix components [20]. This will free some viable cells from the matrix and make a void space for swimming cells. The nutrients released from the dead and lysed cells may support growth of the swimming, dispersing population. The same mechanism may be used to disrupt the matrix at the top of the microcolony to clear a route for dispersing cells. The dispersed cells representing a “second generation” occupy a new surface and re-initiate the cycle as shown in Figure 8A [4].

Many polysaccharides serve as the fibrous and matrix materials to support the structure of plant and animal cellular communities [43]. In this report, we have shown that the Psl polysaccharide also serves as a fibrous, matrix substance to enmesh the bacteria in a biofilm, forming a bacterial ‘tissue’. More interestingly, *P. aeruginosa* undergo cell death and lysis to degrade the Psl matrix in the center of microcolony for future seeding dispersal. This program is similar to development in many higher organisms, including apoptosis and the rapid degradation of matrix components during metamorphosis in amphibians.

In summary, we have shown that the Psl polysaccharide of *P. aeruginosa* forms a matrix, which facilitates surface adherence and maintains biofilm architecture during a biofilm developmental cycle. Moreover, the Psl matrix does not appear to overlap with the eDNA matrix, but appears to coordinate activities to maintain the biofilm structure. Overall, our data indicates that Psl is a key scaffolding component of the *P. aeruginosa* biofilm matrix, a property that likely plays a critical role in *P. aeruginosa* persistence. A better understanding of the biofilm matrix formation and ultra-structure may open up avenues for therapeutics of biofilm-related complications in medical, industrial, and environmental settings.

## Materials and Methods

### Bacterial strains and growth conditions

*P. aeruginosa* strains used in this study are listed in supplemental Table S1. The in-frame Δ*cidAB* and Δ*lrgAB* deletion mutants of *P. aeruginosa* were constructed by an unmarked, non-polar deletion strategy as previously described [18]. *cidAB* and *lrgAB* genes, amplified from *P. aeruginosa* PAO1, were cloned into pUCP18 at *Eco*RI and *Hin*dIII sites, generating plasmid pHP148 (*cidAB*) and pHP149 (*lrgAB*). Unless otherwise indicated, *P. aeruginosa* was grown at 37°C in LB lacking sodium chloride (LBNS) or Jensen's, a chemically defined medium [44]. To induce the transcription of the *psl* operon in strain WFPA801, 0.2 or 2% arabinose was added to Jensen's medium.

### Sequence analysis

Sequence alignment between CidA and LrgA was performed by ClustalW program in *MacVector* 7.0 (ACCELRYS, INC). TMHMM was used for the prediction of trans-membrane helix.

### The flow cell system and microscopy

Unless otherwise indicated, biofilms were grown in Jensen's medium at room temperature in three-channel flow chambers with individual channel dimensions of 1×4×40 mm (Stovall life science, INC). The flow cells were inoculated with ∼10^7^ middle log phase cultures (OD_600_ = 0.5) grown in Jensen's medium. After inoculation, the medium flow was stopped for 1 hour and resumed at a rate of 0.5–0.7 ml/min. Fluorescent-labeled lectin stained biofilms or biofilms after Live/Dead staining (below) were observed and imaged with a Zeiss 510 confocal laser scanning microscope (CLSM) (Carl Zeiss, Jena, Germany). Images were obtained using 63×/1.3 water objective. The software packed with Zeiss LSM510 generated the 3-D images and optical Z-sections. CLSM-captured images were subjected to quantitative image analysis using COMSTAT software [31].

### Lectin and DNA staining

Lectins HHA and MOA were used at a final concentration of 100–200 µg/ml as previously described [21]. For lectin and propidium iodide (PI) double staining, biofilms were first stained by PI (30 µm final concentration) for 15 min. After three washes, PI stained biofilms were stained by FITC-labeled lectins for 2 hours in the dark and observed by CLSM. For lectins and FM4-64 double staining, the lectins-stained surface-attached bacterial cells or biofilm were stained with FM4-64 (1 µm final concentration, Molecular Probes, Invitrogen) for 10 minutes and visualized by CLSM using fluorescein isothiocyanate filter and tetramethylrhodamine isocyanate optical filter (LP650). To stain the surface-attached bacterial cells, overnight culture was diluted to OD_600_ of 0.7∼0.9 and 0.5 ml of the diluted culture was inoculated into a cover glass chamber (Chamber 1.5 German cover glass system, Nalge Nunc International Corp.). The bacterial cells were allowed to attach for 1 hour at room temperature. Non-attached bacterial cells were removed and surface-attached cells were stained by the addition of 0.25 ml of 100 µg/ml lectins. Lectin-stained bacterial cells were either directly observed by CLSM or stained by FM4-64 prior to microscopy. All samples were soaked in PBS buffer during image acquisition. Optical sectioning of lectin-stained bacterial cells was acquired by CLSM with 0.9 µm thickness and 0.45 µm interval. A series of Z-sectioned fluorescent images was deconvoluted by VOLOCITY program (Improvision, INC). The BacLight LIVE/DEAD bacterial viability staining kit (Molecular Probes, Invitrogen) was used to evaluate viability of *P. aeruginosa* in biofilms.

### Cellulase digestion

5.0-mg/ml cellulase (1.38 unit/mg, Sigma) in PBS was used to treat mid-log phase PAO1 cells for 20-h. Treated or untreated samples were used directly in a microtiter dish biofilm assay [30] to test for surface attachment. To visualize the Psl after cellulase treatment, 1.0-ml of induced WFPA801 was allowed to attach to a cover glass chamber for 1-h at room temperature (RT). Cells were washed and the culture was replaced with PBS containing 5.0-mg/ml cellulase. After 16-h incubation at RT and following washing, the glass surface-bound bacteria were stained by HHA-FITC and imaged by CLSM. To test for effects on biofilm formation, cellulase was added to Jensen's media (5.0 mg/ml) and the biofilm was grown under flow conditions for 20-h prior to staining and imaging.

## Supporting Information

Table S1 Strains used in this study.
(0.05 MB DOC)

Video S1 The movie shows a serial optical section of a microcolony taken from its bottom (surface) to the top, which reveals how the Psl matrix encases the bacteria cells in a microcolony. The Psl matrix (red) of 1-day old GFP-tagged WFPA801 biofilm (green) was stained with TRITC-MOA.
(7.86 MB AVI)

Video S2 Time lapse images showing swimming bacteria in the center of a microcolony and a Psl matrix of a 2-days old PAO1 biofilm. Psl matrix was shown in green and bacteria were stained in red by membrane stain FM4-64. The images were taken by every 10 seconds.
(3.99 MB AVI)

Video S3 Time lapse images showing viable swimming cells in the center of a microcolony from a 2-day old WFPA801 biofilm. The biofilm was stained by the viability live/dead stain. The viable cells were stained in green and the dead cells were stained in red. In the movie, only viable cells are shown. The images were taken by every 10 seconds.
(6.39 MB AVI)
