# Supplementary material for: Assembly and Development of the Pseudomonas aeruginosa Biofilm Matrix
Source: PLoS Pathog. 2009 Mar 27;5(3):e1000354. doi: 10.1371/journal.ppat.1000354 (PMC2654510; doi:10.1371/journal.ppat.1000354)
Supplement: Table S1 — Strains used in this study. (0.05 MB DOC) [file ppat.1000354.s001.doc]

Table S1. Strains used in this study

| Strains | Relevant characteristics | Source or reference |
| --- | --- | --- |
| *P. aeruginosa*  PAO1-derived strains |  |  |
| PAO1 | Wild type strain |  |
| WFPA800 | *psl* operon promoter deletion mutant , Δ*psl* | [1] |
| WFPA801 | *psl*-inducible strain, Δ*psl*/P_BAD_-*psl* | [1] |
| WFPA801/pMRP9 | WFPA801 harboring pMRP9, a plasmid expressing GFP constitutively. | [1] |
| WFPA829 | PAO1 Δ*cidAB* | This study |
| WFPA834 | PAO1 Δ*lrgAB* | This study |
| WFPA829/pHP148 | Δ*cidAB* harborig pHP148, a plasmid expressing *cidAB* constitutively, Amp^r^ | This study |
| WFPA834/pHP149 | Δ*lrgAB* harborig pHP149, a plasmid expressing *lrgAB* constitutively, Amp^r^ | This study |
| *rpoN* mutant | PAO1 Δ*rpoN*, | [2] |
| *fliMpilA* double mutant | PAO1 Δ*fliMΔpilA*, Gm^r^,Tc^r^ | [3] |

1. Ma L-Y, Jackson K, Landry RM, Parsek MR, Wozniak DJ (2006) Analysis of *Pseudomonas aeruginosa* conditional Psl variants reveals roles for the Psl polysaccharide in adhesion and maintaining biofilm structure postattachment. J Bacteriol 188: 8213-8221.

2. Garrett E, Wozniak DJ (1999) Negative control of flagellum synthesis in *Pseudomonas aeruginosa* is modulated by the alternative sigma factor AlgT (AlgU). J Bacteriol 181: 7401-7404.

3. Allesen-Holm M, Barken KB, Yang L, Klausen M, Webb JS, et al. (2006) A characterization of DNA release in *Pseudomonas aeruginosa* cultures and biofilms. Mol Microbiol 59: 1114-1128.
